# Experimental Study on the Performance of a Stable Foam System and Its Application Effect Combined with Natural Gas in Natural Foamy Oil Reservoirs

**DOI:** 10.3390/polym17222966

**Published:** 2025-11-07

**Authors:** Jipeng Zhang, Yongbin Wu, Xingmin Li, Chao Wang, Pengcheng Liu

**Affiliations:** 1 School of Energy Resources, China University of Geosciences (Beijing), Beijing 100083, China; 18152696912@163.com; 2 State Key Laboratory of Enhanced Oil and Gas Recovery, Research Institute of Petroleum Exploration and Development, PetroChina, Beijing 100083, China; wangchao21@petrochina.com.cn; 3 Research Institute of Petroleum Exploration and Development, PetroChina, Beijing 100083, China; lxingmin@petrochina.com.cn

**Keywords:** natural foamy oil reservoirs, natural gas–chemical synergistic huff and puff, stable foam plugging system, polymer–surfactant composite, enhanced oil recovery

## Abstract

Reservoirs in the Orinoco Heavy Oil Belt, Venezuela, typically hold natural foamy oil. Gas liberation during depletion leads to a sharp increase in viscosity, adversely impacting development efficiency. Therefore, this paper proposes a natural gas (CH_4_)–chemical synergistic huff-and-puff method (CCHP). It utilizes the synergism between a stable foam plugging system and natural gas to supplement reservoir energy and promote the generation of secondary foamy oil. To evaluate the performance of 20 types of foam stabilizers (polymers and surfactants), elucidate the influence on production and properties of key parameters, and reveal the flow characteristics of produced fluids, 24 sets of foam performance evaluation tests were conducted using a high-temperature foam instrument. Moreover, 15 sets of core experiments with production fluid visualization were performed. The results demonstrate that, in terms of individual components, XTG and HPAM-20M demonstrated the best foam-stabilizing performance, achieving an initial foam volume of 280 mL and a foam half-life of 48 h. Conversely, the polymer–surfactant composite of XTG-CBM-DA elevated the initial foam volume to 330 mL while maintaining a comparable half-life, further enhancing the performance of foaming capacity for a stable foam system. For further application in the CCHP, oil production shows a positive correlation with both post-depletion pressure and chemical agent concentration; however, the foam gas–liquid ratio (GLR) exhibits an inflection point, with the optimal ratio found to be 1.2 m^3^/m^3^. During the huff-and-puff process, the density and viscosity of the produced oil decrease cycle by cycle, while resin and asphaltene content show a significant reduction. Furthermore, visualization results reveal that the foam becomes finer, more stable, and more uniformly distributed under precise parameter control, leading to enhanced foamy oil effects and improved plugging capacity. Moreover, the foam structure transitions from an oil-rich state to a homogeneous and stable configuration throughout the CCHP process. This study provides valuable insights for achieving stable and sustainable development in natural foamy oil reservoirs.

## 1. Introduction

The Orinoco Heavy Oil Belt in Venezuela represents a globally typical natural foamy oil reservoir with recoverable reserves of up to 460 × 10^8^ t [1,2]. Its dead oil viscosity generally ranges between approximately 5000 and 25,000 mPa·s, with a high asphaltene and resin content (15~25%), as well as a low gas–oil ratio (GOR < 20 m^3^/m^3^) in the reservoir [3]. Its unique characteristics include the spontaneous formation of stable in situ foamy oil under high-pressure conditions, where crude oil and dissolved gas coexist. This foamy oil differs from conventional gas–liquid biphasic foam, manifesting as a pseudo-single-phase system where microbubbles are uniformly dispersed within ultra-heavy oil [4,5,6]. Therefore, the foamy oil exhibits distinctive rheological properties, meaning that it exhibits significantly reduced viscosity compared to degassed crude oil at equivalent temperatures under reservoir conditions [7]. However, although foamy oil improves production performance through the solution gas drive in heavy oil, less than 15% of the original oil in place (OOIP) can be recovered [8,9]. This phenomenon constitutes both the production advantage of using natural energy and the core contradiction limiting its efficient development.

Natural foamy oil, which relies on the gradual release of dissolved gas and the stabilization of asphaltene–gas interfaces, traditionally exhibits a slow pressure decline rate [6,10]. When the reservoir pressure falls below the bubble-point pressure, the gas–oil ratio does not increase rapidly. However, in recent years, rapid pressure depletion in the Orinoco Belt has been observed, causing gas bubbles in the foamy oil to expand and coalesce abruptly [11,12,13]. This results in a sharp increase in viscosity and a significant reduction in fluid mobility. Therefore, maintaining in situ foamy oil stabilization constitutes a critical requirement for ensuring the sustainable development of oil production. Gas injection has proven successful in some foamy oil reservoirs [14]. Previous studies have investigated the potential of natural gas injection following primary production in the Orinoco Belt [15,16]. The results revealed that the dissolution of natural gas into the oil leads to crude oil swelling, viscosity reduction, and the generation of artificial foamy oil. It was found that the huff-and-puff process exhibits greater potential compared to displacement methods.

However, the issue of gas channeling consistently accompanies gas injection development processes, including those involving natural gas injection in natural foamy oil reservoirs [17,18,19,20]. To address these challenges, a method known as the CH_4_–chemical synergistic huff-and-puff method (CCHP) has been proposed. This technique combines a stable foam system with natural gas. The stable foam system, composed of foaming agents and foam stabilizers, interacts with the injected gas to generate highly stable foam [21,22]. This approach effectively mitigates gas channeling and reservoir pressure depletion, promotes sufficient dissolution of natural gas, and improves sweep efficiency. Through the synergistic effect of CH_4_ and the stable foam system, the CCHP enhances the generation of secondary foamy oil within the reservoir, enabling efficient gas utilization and improved crude oil recovery.

The essence of the CCHP lies in restructuring the formation, migration, and stabilization processes of foamy oil through multi-scale gas–liquid interactions within the reservoir. The role of the stable foam system is particularly critical in this process, as it determines the performance and longevity of the generated foam, especially in high-salinity and high-asphalt-content reservoir environments [23,24]. These systems are diverse in type, including surfactants, polymers, nanoparticles and their mixtures; therefore, the selection of an optimized stable foam system is essential [25,26,27,28,29,30]. Moreover, it is crucial for the CCHP to elucidate production performance and EOR mechanisms in natural foamy oil reservoirs, as these factors directly impact both the feasibility and sustainability of the development.

The formation of a stable foam system is a complex process. Foam is inherently a thermodynamically unstable system, and its decay primarily involves liquid film drainage, bubble coalescence, and gas diffusion [31]. The core mission of foam stabilizers is to delay or prevent these decay processes through various physicochemical mechanisms. Surfactants are the most fundamental foaming and foam-stabilizing agents, and they can generate foam by significantly reducing surface tension [32]. Numerous studies have demonstrated that this leads to a significant enhancement in oil recovery [32,33,34,35]. Crucially, when the liquid film is locally thinned by external impact, the surfactants adsorbed at the interface generate a concentration gradient and enable self-healing of the liquid film via the Marangoni effect [36]. Polymers significantly retard liquid film drainage and enhance the mechanical strength of the foam by increasing the viscosity of the liquid phase and forming a viscoelastic interfacial film [37,38]. Nanoparticles are adsorbed at the interface to form a dense “armor layer of particles”, which suppresses bubble coalescence through physical steric hindrance. Their unique solid barrier structure imparts exceptional long-term stability and environmental tolerance to the foam [39]. In addition, other materials such as ordinary solid particles, biosurfactants, protein-based foam stabilizers, and some water-insoluble aliphatic compounds also exhibit good foam-stabilizing properties [40]. However, their application is limited due to issues such as performance effectiveness and economic cost.

However, the foam-stabilizing performance of a single agent is inherently limited. The excessive addition of polymers, for example, can impair foam generation, whereas a low concentration results in poor foam stability [41]. To resolve this issue and further enhance oil recovery, it is necessary to develop a composite formulation to establish more effective stable foam systems, such as polymer–surfactant composites [42]. These can further enhance foam stability without compromising foam generation by effectively reinforcing the liquid film and interacting with the natural surfactants present in heavy oil [43].

Specific crude oil and reservoir properties dictate the optimal stable foam system and its parameters. These factors, in turn, determine the effectiveness of production enhancement and the underlying mechanisms. Consequently, in this paper, based on the MPE3 block in the Orinoco Heavy Oil Belt, Venezuela, 24 foam stability evaluations of single agents and composites were conducted using a high-temperature foam analyzer, complemented by 15 groups of CCHP core tests performed with a production visualization unit. These experimental results systematically elucidated performance variations among foam stabilizers, established key parameters controlling CCHP oil recovery, and clarified the properties of produced oil along with flow mechanisms in the produced fluids throughout the production process. This study provides practical insights into the feasibility and effectiveness of the CCHP in natural foamy oil reservoirs.

## 2. Experimental Methodology

The MPE3 block is characterized by the formation of a water environment with high salinity and the formation of crude oil with a high asphalt content. The high concentration of ions along with asphalt adsorption can destabilize the foam. Therefore, key experiments, including examination of the foaming performance and the CCHP, using 1D core, were conducted to effectively screen salt-resistant and oil-resistant stable foam systems and to reveal the effectiveness of the CCHP on EOR.

### 2.1. Materials

1. Water: Based on the in situ properties of water from the MPE3 block, simulated formation water is replicated using CaCl_2_, MgCl_2_, FeCl_2_, NaCl, and NaHCO_3_ at quantities of 0.773, 0.602, 0.193, 17.285, and 2.685 g/L, respectively.
2. Oil: Heavy oil produced from the MPE3 block was prepared for the experiments using SARA, as listed in Table 1, exhibiting a dead oil viscosity and density of 35,600 mPa·s and 0.96 g/cm^3^ at 50 °C. Long-term storage caused evaporation of light components in the oil samples. We added light hydrocarbons to replicate crude oil, based on the composition analysis listed in Table 2.
3. Gas: Methane (CH_4_) with a purity of 99.9% was used.
4. Basic foaming agents: NKS and SX-117 from Macklin Biochemical Technology Co., Ltd., Shanghai, China.
5. Foam stabilizers: Polyacrylamide, Dodecanol, Hexadecanol, modified silicone resin polyether, modified silicone resin polyether emulsion, modified acrylic polymer, acrylic polymer film-forming agent, Xanthan gum, acrylic polymer, Hydroxypropyl methyl cellulose type II, Hydroxyethyl cellulose, Polyethylene glycol, Polyvinylpyrrolidone, Nano-silica. All the agents are from Macklin Biochemical Technology Co., Ltd., Shanghai, China.

### 2.2. Experimental Apparatus

#### 2.2.1. High-Temperature Foam Instrument

**Figure 1:**
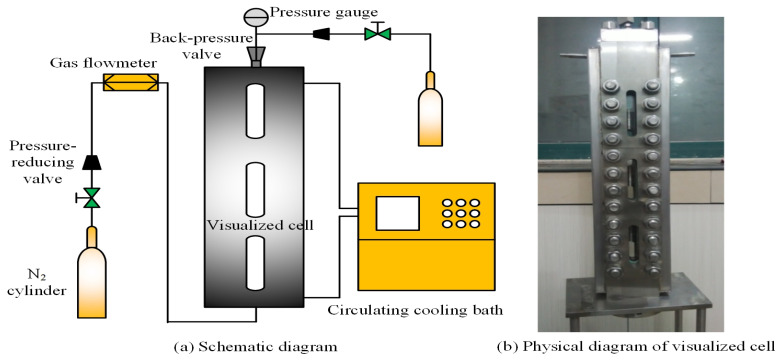
High-temperature foam instrument.

Figure 1 displays the high-temperature foam instrument manufactured in Jiangsu, China, which primarily consists of a CH_4_ cylinder, a gas flowmeter, a visualized cell with a rotor, a back-pressure regulation (BPR), a circulating cooling bath, and a pressure gauge. The gas flowmeter measures injection volume, while the variations in foam volume during the process are recorded through observation and measurement of the visualized cell. BPR controls the cell pressure, and the circulating cooling bath is employed for rapid temperature regulation.

#### 2.2.2. One-Dimensional Core Model with Visualization Unit

**Figure 2:**
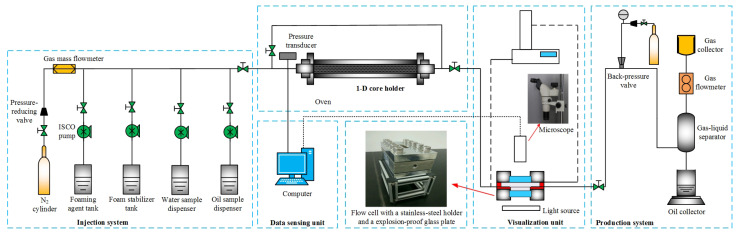
Schematic diagram of the orthogonal experimental apparatus.

Figure 2 depicts a one-dimensional (1D) model consisting of five units used for conducting core experiments with the CCHP to reveal the influence of different parameters; this model is manufactured in Jiangsu, China.

1. Injection unit: This consists of a CH_4_ cylinder, an oil sample dispenser, a chemical tank, two pressure-reducing valves, a gas mass flowmeter, and three ISCO pumps. The oil sample dispenser is used to prepare foamy oil sample consistent with the reservoir condition. The pressure-reducing valve is used to control the injection pressure, and the ISCO pumps are used to inject fluids.
2. One-dimensional core holder: This is composed of an oven with a core holder diameter of 2.5 cm and a length of 50 cm. The oven is heated to control the temperature of the huff and puff. The core holder is filled with the formation core to simulate the actual reservoir. It has an average porosity of 42.2%, an average permeability of 7.3 μm^2^, and an initial oil saturation of 97.2.
3. Visualization unit: This consists of a thickened explosion-proof glass plate, a flow cell, a stainless-steel holder, an annular pressure pump, and a microscope with a light source. The flow behavior was observed in flow cells using the microscope.
4. Production unit: This consists of a vacuum pump, a back-pressure regulator (BPR), a CH_4_ cylinder, a pressure-reducing valve, a pressure gauge, a gas–liquid separator, a gas flowmeter, an oil collector, and a gas collector. The gas–liquid separator is used to separate produced oil and gas. The vacuum pump is used for saturation, and the BPR is used to adjust the production pressure.
5. Data-sensing unit. This is composed of a pressure transducer and a computer, which can transmit and record pressure data in real time.

### 2.3. Experimental Scenarios

#### 2.3.1. Foam Performance Tests

**Figure 3:**
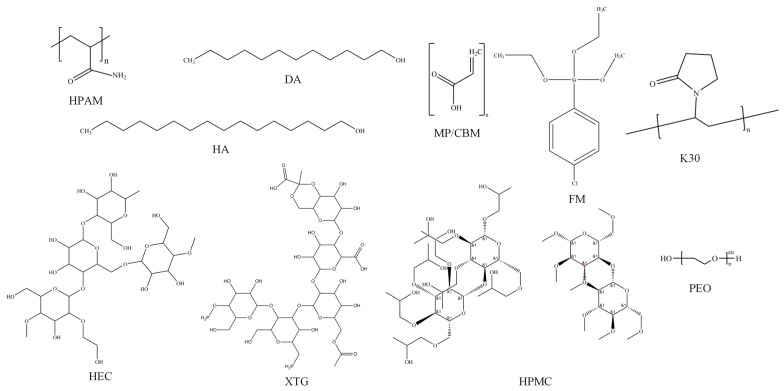
Proposed chemical structure of foam stabilizers.

Foam stabilizers can be categorized into two types based on their modes of action in improving foam stability, which are the mechanical strength enhancement of liquid films and the increased viscosity of aqueous solutions. Therefore, as summarized in Table 3 with the proposed chemical structure given in Figure 3, this study collected 20 types of foam stabilizers, predominantly polymers, along with a small number of surfactants and nanoparticles, and 24 sets of experimental schemes were designed to evaluate the performance of different components and their composites.

The foam stabilizer was incorporated at a mass fraction of 1.0%, with the base foaming agent comprising a surfactant mixture of NKS and SX-117 with a mass fraction of 3%. The stable foam system for measurement was formulated using simulated formation water with a total volume of 100 mL. This also contained 50 mL of crude oil to establish oil saturation at 50%. The performance of the foam stabilizer was compared and optimized under both high-salinity and high-oil-saturation reservoir conditions.

#### 2.3.2. One-Dimensional Core Experiments of the CCHP

The core experiments with production visualization were conducted to elucidate the improved production performance and enhanced recovery efficiency of the CCHP in a natural foamy oil system, along with flow characterization of the foam. Several critical parameters warrant investigation due to their practical significance in field applications, particularly regarding post-depletion pressure, the gas–liquid ratio (GLR) of foam, chemical concentration, and injection condition.

Table 4 lists the specific design of 15 sets of experiments to clarify the influence of these parameters on development. Herein, the concentration of chemical agents was determined based on previous research findings. In their study on surfactant flooding in the MPE3 block, Li et al. [44] and Sun et al. [45] observed the adsorption loss of chemicals during the process. Consequently, they set the concentrations of the surfactant and the foam stabilizer at relatively high levels of 1.0–3.0% and 1.0%, respectively, achieving favorable results. Therefore, this study further refines the chemical concentrations to obtain a more comprehensive impact trend.

### 2.4. Experimental Procedure

#### 2.4.1. Foam Performance Tests

The following is a brief description of the experimental steps.

1. Chemical preparation: Foam agent-stabilizer solutions are prepared in beakers, where stabilizers are finally dispersed into the solution to prevent micelle formation, followed by incubation in a 55 °C thermostatic water bath. Oil samples are prepared using a dispenser by incorporating the missing light hydrocarbons with stock oil. Finally, BPR is adjusted at 3.0 MPa, while cell temperature is maintained at 55 °C.
2. Co-injection: Foam agent-stabilizer solutions and oil samples are co-injected into the visualization cell; CH_4_ is then injected to increase the cell pressure to 3.0 MPa.
3. Mixing: The cell rotor is set to rotate at a low speed of 400~500 r/min for 3.0 min, ramping to 1500 r/min sustained shearing for 6.0 min.
4. Record: The variations in drainage height and foam height are continuously recorded at designated intervals.

#### 2.4.2. One-Dimensional Core Experiments of the CCHP

The following is a brief description of the experimental steps.

1. Preparation: Foaming agent-stabilizer solutions are prepared at specified concentrations according to the experimental scenarios, where the corresponding foam stabilizer was formulated based on the performance evaluation tests. Meanwhile, oil samples are prepared using a dispenser by incorporating the missing light hydrocarbons with stock oil. The oven is adjusted to 55 °C to match the reservoir temperature, and the BPR is adjusted to 8.45 MPa to match the reservoir pressure.
2. Water saturated: The core is saturated with water at a constant injection rate. The core properties are measured, and the results are shown in Table 5.
3. OOIP and depletion: The core is vacuumized to saturate water; the oil sample is then injected to establish the original oil in place (OOIP), shown in Table 5, which closely approximates the in situ oil saturation measured from the original core. After being aged for 24 h, depressurization-depletion production is initiated by gradually reducing the pressure to the experimental scenarios.
4. CCHP: CH_4_ and the foaming agent-stabilizer solutions are alternately injected into the core at the GLR set in the specific scenarios until the pressure reaches 8.45 MPa. Then, all valves are closed to soak the wellbore for 10.0 min. Finally, the BPR is adjusted to the set production pressure (bottom hole pressure) for production. The first cycle of throughput ends when no oil is produced.
5. Visualization: When the water cut in the produced fluid exceeds 98%, the foam morphology in the produced liquid is recorded, as it can effectively reflect the in situ performance of foam in the reservoir.
6. Multiple cycles: Repeat step 4 to complete 8 cycles of the CCHP.

## 3. Results and Discussing

### 3.1. Optimization of Foam Stabilizers and Performance of Stable Foam System

#### 3.1.1. Single Component

**Figure 4:**
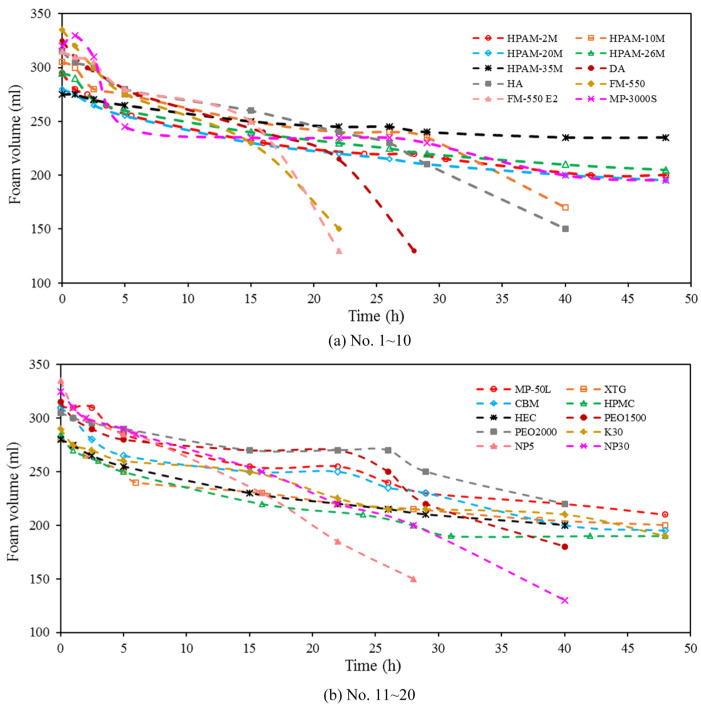
Foam volume of stable foam system with different types of foam stabilizers.

**Figure 5:**
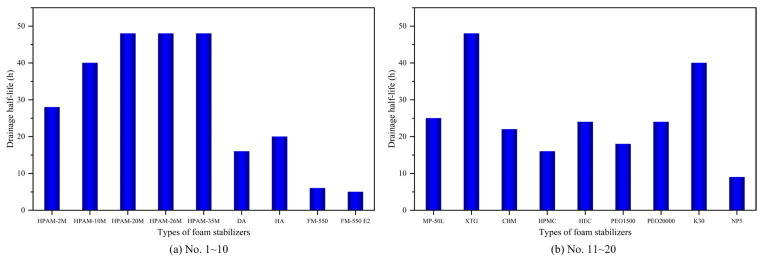
Drainage half-life of stable foam system with different types of foam stabilizers.

Under the presence of different foam stabilizers, the system exhibits distinct effectiveness on foaming and foam stabilizing. Figure 4 and Figure 5 display the foam volume and drainage half-life of a stable foam system with different types of foam stabilizers. As shown, in the 20 foam stabilizers with a single component, surfactants further enhanced the initial foam volume due to their ability to reduce interfacial tension, while polymers demonstrated superior comprehensive foam-stabilizing performance compared to surfactants and inorganic nanoparticles.

Notably, due to the superior oil tolerance and salt resistance, XTG exhibited the optimal foam stability, with a half-life exceeding 48 h and a relative foam volume of 280 mL; HPAM-2M/20M exhibited a half-life of 28/48 h and a foam volume of 295/280 mL. Moreover, despite generating a relatively high initial foam volume of around 335 mL, NP5 and FM-550, exhibited poor stability performance, with a half-life of less than 10 h. Overall, foam-stabilizing performance improves as the molecular weight increases, although at the expense of reduced solubility and compromised foaming capability.

As water-soluble polymers, HPAM and XTG primarily interact with surfactant molecules through hydrophobic interactions. They form soft matter clusters with surfactants, wherein a minority of polymer chains bind to the clusters via hydrophobic association, while the majority extend as loops into the aqueous phase. Under the combined effects of electrostatic forces, hydrogen bonding, and Van der Waals forces, the polymers assemble into a giant three-dimensional spatial network along with the surfactant. This structure imparts exceptional viscosity enhancement and shear resistance to the entire system.

#### 3.1.2. Polymer–Surfactant Composite

**Figure 6:**
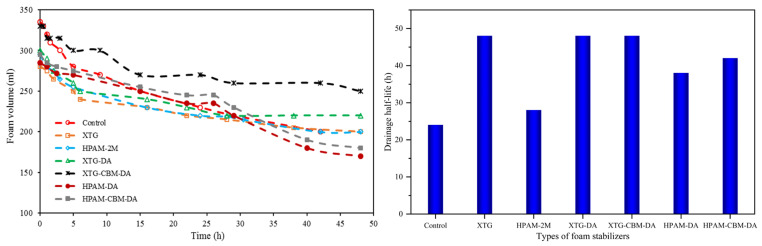
Foam volume and drainage half-life of stable foam system with the polymer–surfactant composite.

However, a single foam stabilizer cannot achieve the balanced optimization of both foam generation and foam stability. The excessive addition of polymers impairs foaming capacity, while insufficient amounts result in poor foam stability. Figure 6 thus shows the foam volume and drainage half-life of a stable foam system, along with the foam stabilizers of the polymer–surfactant composite.

As shown in Figure 6, the composites universally enhanced performance relative to single-component agents. Specifically, the foam volume curves for composites (HPAM-DA, HPAM-CBM-DA, XTG-CBM-DA) showed a distinct stepwise improvement, directly reflecting enhanced foam stability. This behavior results from the ability of the composites to effectively retard the coalescence and drainage of foam lamellae.

XTG-CBM-DA and HPAM-CBM-DA showed outstanding performance, with initial foaming volumes of 330 mL and 295 mL and drainage half-lives of 48 h and 42 h, respectively. Regarding foaming capacity, the polymer–surfactant composite of XTG-CBM-DA exhibited a 50 mL increase in initial foam volume compared to XTG, owing to the effective improvement of foaming performance by the incorporated surfactant. In terms of foam stability, the polymer–surfactant composite of HPAM-CBM-DA significantly extended the foam half-life, with a 14 h increase in drainage half-life compared to HPAM. XTG-CBM-DA possessed a superior foam-stabilizing capacity than the average level of a single component; this was also optimal in the composites. Therefore, the polymer–surfactant composite improves the comprehensive performance of foaming capacity and foam stability.

For the polymer–surfactant composite, the polymer increases the viscosity of the dispersed phase through its inherent high molecular weight, thereby reducing fluid mobility. Meanwhile, polar head groups of surfactants interact synergistically with the hydrophilic moieties of polymers, adsorbing at the interface to enhance interfacial film strength and viscoelasticity. This effectively suppresses oil droplet coalescence, demonstrating significant stability improvements in high-salinity and oil-saturated reservoir environments.

Substantial research has been conducted on stable foam systems. Ahmed et al. [46] reported that HPAM achieved a foam volume of 277.1 mL and a foam half-life of 0.52 h, while Chen et al. [47] found that DYG exhibited a foam volume of 415 mL and a liquid drainage half-life of 0.88 h. Similarly, Adil et al. [48] investigated polymer–surfactant stabilized foam systems (SC-XTG and SDS-XTG), reporting foam half-lives of 3.1 h and 1.2 h, along with drainage half-lives of 0.3 h and 0.4 h, respectively. Compared with the present study, the differences in foam volume and half-life are primarily attributable to variations in salinity and chemical concentration. Therefore, adjusting the chemical formulation and ratio according to specific reservoir conditions is essential to achieve optimal foam performance.

### 3.2. Production Performance and Parameters Influence of the CCHP

#### 3.2.1. Pressure After Natural Depletion

**Figure 7:**
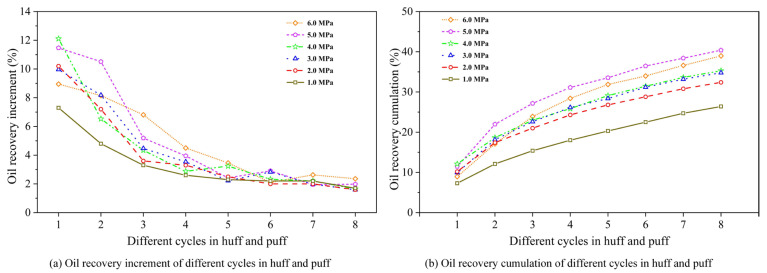
Oil recovery variation under different post-depletion pressures.

Figure 7 illustrates the oil recovery variation under different post-depletion pressures, including incremental and cumulative recovery per cycle. From Figure 6, it is evident that a higher post-depletion pressure leads to a greater oil recovery during the huff-and-puff process, though the incremental gain diminishes beyond 3.0 MPa. Notably, lower initial pressures during the transition from natural depletion to huff and puff correlated with reduced incremental recovery per cycle, particularly after the second cycle where this positive correlation became statistically significant. By the 8th cycle, differences in incremental recovery across pressure schemes diminished substantially.

The positive correlation between post-depletion pressure and incremental recovery was absent in the two initial cycles due to the original dissolved gas. Higher post-depletion pressure trapped more original dissolved gas in the reservoir, which was then liberated during subsequent huff-and-puff cycles. This sharply increased the gas–liquid ratio in the initial two cycles, impairing production and reducing incremental recovery at 5.0–6.0 MPa by 2.0–3.0% compared to 4.0 MPa in the first cycle. However, at the release and production of this original dissolved gas, its negative effect on the elevated gas–liquid ratio was gradually outweighed by the enhanced viscosity reduction at higher pressures, thereby restoring the positive correlation in the later four cycles.

The underlying mechanism revealed that lower post-depletion pressures intensified crude oil degassing, reducing the dissolved gas content and consequently elevating oil viscosity. This resulted in diminished cyclic oil production and lower recovery efficiency, which were especially pronounced during the initial four cycles. Inter-cycle production differentials narrowed significantly after four cycles. Thus, disparity in the CCHP primarily manifests during the high-pressure stage where sufficient dissolved gas remains.

#### 3.2.2. Gas–Liquid Ratio of Foam

**Figure 8:**
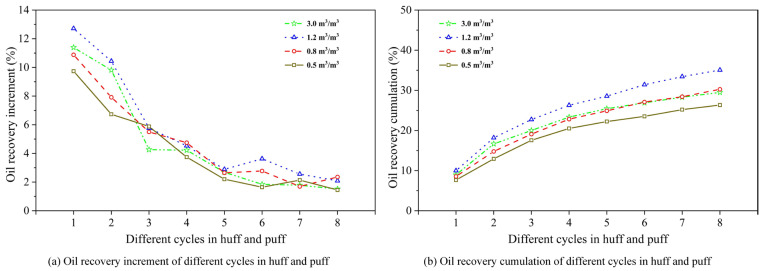
Oil recovery variation for different gas–liquid ratios.

Figure 8 illustrates the oil recovery variation under different GLRs, including incremental and cumulative recovery per cycle.

From Figure 8, under high-GLR conditions, the first two cycles in the CCHP demonstrated superior foam generation efficacy, yielding higher cyclic recovery increment compared to low-GLR scenarios. However, subsequent cycles exhibited significant production fluctuations with minimal inter-cycle variation. Cumulative recovery analysis revealed an inflection point in GLR-dependent recovery performance, where cycles at GLRs of 0.8 and 3.0 m^3^/m^3^ showed markedly lower oil recovery than those at the optimal GLR of 1.2 m^3^/m^3^.

Moderate GLR elevation enhanced CH_4_ foam formation. The generated foam effectively retarded hydrocarbon gas liberation from crude oil, thereby suppressing degassing. Consequently, foam generation is critical for enabling secondary foamy oil production.

#### 3.2.3. Concentration of Chemical Agent

**Figure 9:**
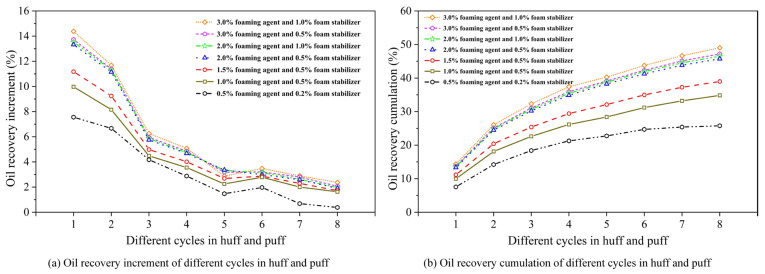
Oil recovery variation for different chemical concentrations.

Figure 9 shows the oil recovery variation under different chemical concentrations, including incremental and cumulative recovery per cycle. From Figure 8, it can be seen that as the concentration of the foaming system decreased from 3.0% foaming agent and 1.0% stabilizer, the cyclic recovery increment of the CCHP progressively declined, with an inflection point observed at 2.0% foaming agent and 0.5% stabilizer. Particularly at concentrations of 0.5% foaming agent and 0.2% stabilizer, oil recovery performance is significantly diminished in the CCHP. This reduction stemmed from insufficient concentrations inhibiting foamy oil regeneration. Conversely, increasing the concentrations of both the foaming agent and the stabilizer enhances the oil recovery factor.

These results demonstrated that optimal foaming agent concentrations promoted secondary foamy oil regeneration, thereby extending the foamy oil production period and improving the efficacy of the CCHP. Considering the influence of formation adsorption on chemical concentration, high-concentration injection during early cycles was recommended.

### 3.3. Physical Properties of Produced Oil

#### 3.3.1. Phase Density and Viscosity of Produced Oil

**Figure 10:**
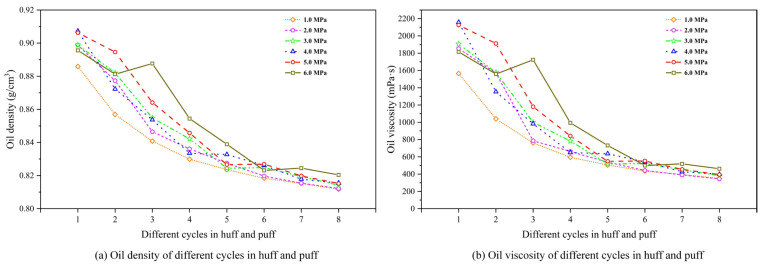
Oil density and viscosity variation under different post-depletion pressures.

Variations in the density and viscosity of produced fluids reflect changes in oil quality, indicating the effectiveness of foaming and stabilizing agents in natural foamy reservoirs. Figure 10 illustrates the variation in oil density and viscosity under different depletion pressures.

As shown in Figure 10a,b, within each experimental scheme, the density and viscosity of produced oil gradually decrease with increasing cycles in the CCHP, ranging from 0.91 to 0.81 g/cm^3^ and from 2200 to 200 mPa·s. This is particularly pronounced during the initial four cycles. This reduction stems from declining crude oil production per cycle while the dissolved gas content remains relatively stable, resulting in lower oil-phase density. Additionally, comparative analysis has revealed that higher injection transfer pressure yields slightly greater oil-phase density and viscosity than lower pressure during the initial four cycles. This correlation confirms enhanced crude oil production following high-pressure injection in early-stage operations.

However, at a depletion pressure of 6.0 MPa, unusual decreases in oil density and viscosity were observed during the first and second cycles. This phenomenon can be attributed to the high post-depletion pressure, which limited the release and production of original dissolved gas, causing a greater portion of the original dissolved gas to remain in the solution within the crude oil in the reservoir. Consequently, during the initial two cycles of the huff-and-puff process, the production of substantial amounts of this dissolved gas within the crude oil led to a significant reduction in oil viscosity and density. A similar trend was observed in the first cycle of other experimental scenarios (5.0 MPa).

**Figure 11:**
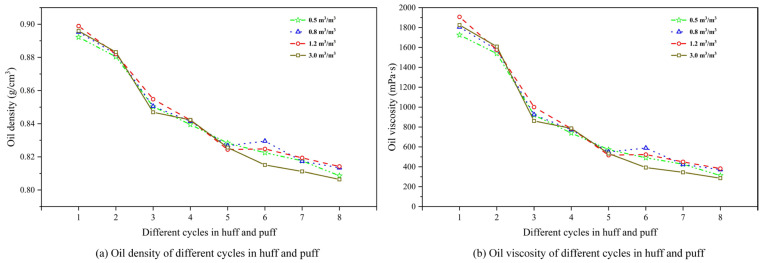
Oil density and viscosity variation for different gas–liquid ratios.

Figure 11 shows the evolution of oil density and viscosity for different GLRs. From Figure 10a,b, within a fixed GLR, it can be seen that the density and viscosity of produced oil progressively decrease with successive cycles in the CCHP, ranging from 0.90 to 0.80 g/cm^3^ and 2200 to 200 mPa·s. They exhibit a particularly pronounced decline between the second and third cycles. This pattern confirms that the stable foam system achieves optimal foam generation and oil production performance during the initial two cycles.

Subsequent cycles yield diminished crude oil volumes while the dissolved gas content remains relatively stable, collectively driving reductions in both the density and viscosity of the produced oil phase. Meanwhile, oil-phase properties vary significantly across different GLRs. During the first two cycles, higher GLRs correlated with increased oil-phase density and viscosity, with the peak at a ratio of 1.2 m^3^/m^3^, indicating maximal oil production at this optimal ratio. Further, elevating the ratio to 3.0 m^3^/m^3^ reduced the oil production rate, manifested through a measurable decline in oil-phase density.

**Figure 12:**
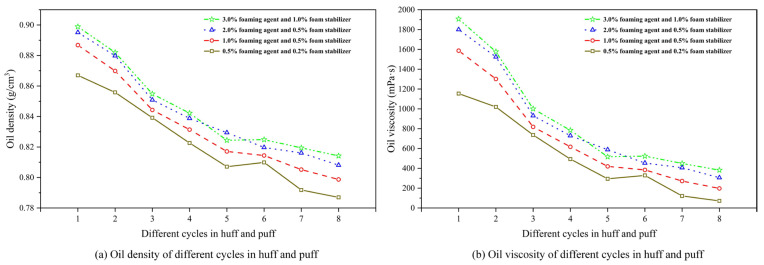
Oil density and viscosity variation for different chemical concentrations.

Figure 12 shows the evolution of oil density and viscosity for different chemical concentrations. From Figure 12a,b, it can be seen that for each chemical agent concentration, both the density and viscosity of produced oil progressively decrease with increasing cycles in the CCHP, ranging from 0.90 to 0.78 g/cm^3^ and from 2000 to 50 mPa·s. They exhibit a particularly pronounced decline at the transition to the third cycle following the initial two cycles. Meanwhile, both the density and viscosity in the oil phase diminished with reduced concentrations of foaming and stabilizing agents. This trend arose because lower agent concentrations inhibit the regeneration of secondary foamy oil, thereby increasing produced gas volume and impairing the development efficiency of the CCHP, leading to a reduction in overall viscosity and density.

The concentration of chemical agents is the most significant parameter affecting oil properties. Unlike post-depletion pressure, the influence of chemical concentration persists throughout the entire huff-and-puff process and is relatively uniform across different cycles. Higher chemical concentrations enhance oil displacement efficiency, but are accompanied by an increase in both oil viscosity and density. Consequently, careful attention must be paid to oil flow in the wellbore during production.

#### 3.3.2. SARA Variation in Produced Oil

**Figure 13:**
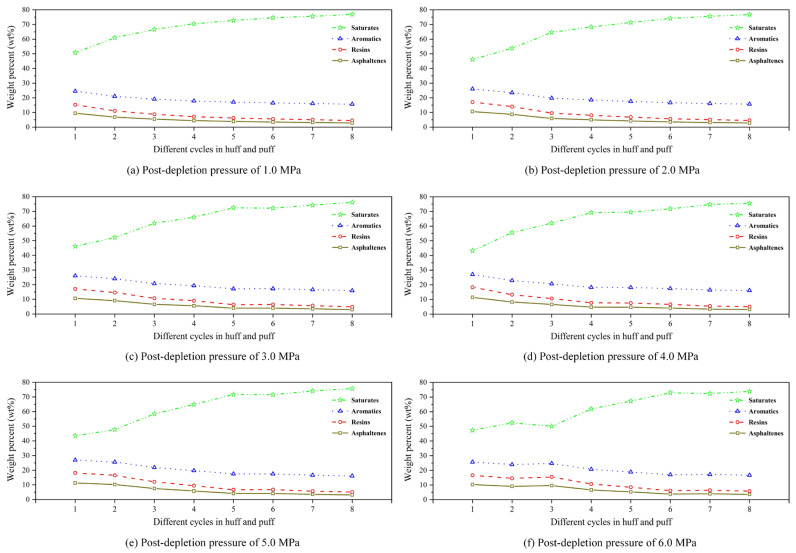
SARA variation under different post-depletion pressures.

Figure 13 shows the SARA variation under different post-depletion pressures. From Figure 13a–f, within each scheme, it can be seen that the saturate content in the produced oil phase progressively increases with successive cycles of the CCHP, exhibiting a marked rise during the first four cycles. Conversely, the content of aromatics was shown to decrease marginally, while that of resins and asphaltenes dropped significantly to below 10%. This compositional shift occurred because oil production volume diminished cyclically, whereas dissolved gas output remained relatively constant, leading to an increasing proportion of light hydrocarbon solvents in the produced mixture.

Furthermore, oil-phase composition varies distinctly with different post-depletion pressures. During the initial four cycles, low post-depletion pressure yielded a higher saturate content and a lower asphaltene content compared to the high post-depletion pressure. This confirms that low post-depletion pressure produces less crude oil than high post-depletion pressure in early-stage cycles.

At a post-depletion pressure of 6.0 MPa, an anomalous point occurred in the third cycle, showing decreased contents of saturates but increased contents of aromatics, resins, and asphaltenes. This shift, consistent with anomalies in recovery, density, and viscosity, resulted from the rapid production of substantial amount of original dissolved gas during the first two cycles. This resulted in the production of oil that was enriched with lighter components, thereby increasing the content of saturates. By the third cycle, the original dissolved gas was largely depleted, causing the observed drop in the content of saturates.

**Figure 14:**
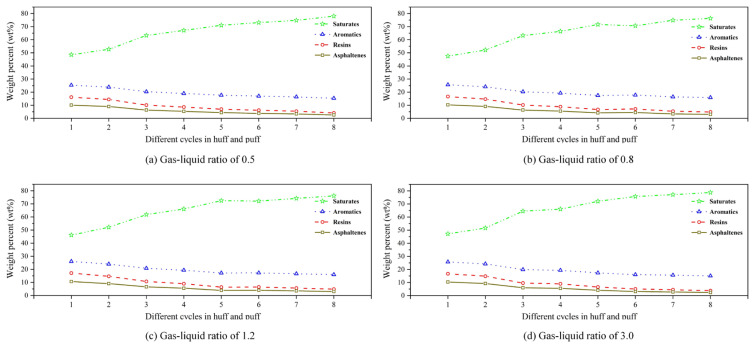
SARA variation for different gas–liquid ratios.

Figure 14 displays the SARA variation for different GLRs. From Figure 14a–d, within a fixed GLR scheme, the saturate content in the produced oil phase progressively increases with successive cycles in the CCHP, exhibiting a significant rise during the first two cycles. Conversely, the aromatic content decreased marginally, while that of resins and asphaltenes declined substantially.

Meanwhile, compositional differences emerged across varying GLRs. In the initial two cycles, stable foam systems with lower GLRs yielded a slightly higher saturate content and lower asphaltene content compared to those with higher ratios, indicating that higher-GLR foam produced greater crude oil volumes than lower-ratio foam during early-stage operations.

**Figure 15:**
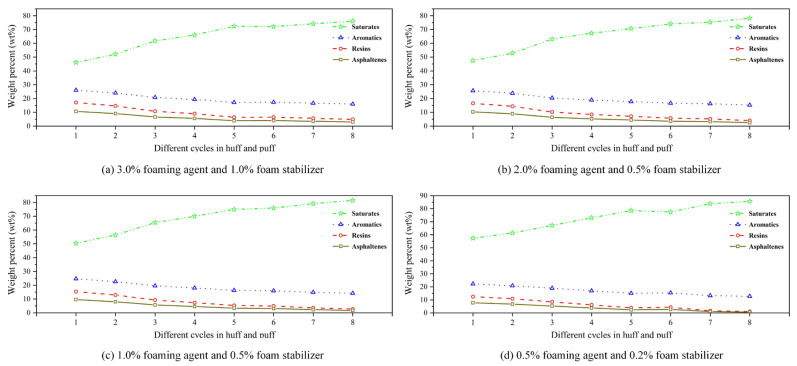
SARA variation for different chemical concentrations.

Figure 15 displays the SARA variation for different gas–liquid ratios. From Figure 15a–d, from each chemical agent concentration, the content of saturates in the produced oil phase progressively increases with successive cycles in the CCHP, exhibiting a pronounced incresae during the first four cycles. Conversely, the content of aromatics decreased marginally, while that of resins and asphaltenes declined significantly to around 10%.

Meanwhile, compositional variations emerged across different chemical concentrations. Across all cycles, schemes with higher chemical agent concentrations yielded slightly lower saturate content and higher asphaltene content compared to those with lower concentrations. This trend indicates that higher chemical agent concentrations enhance crude oil production throughout all cycles relative to lower-concentration systems.

### 3.4. Interplay Between Production Performance and Oil Properties

A close relationship exists between recovery performance and the variation in the SARA composition of produced oil, forming a clear causal chain through fluid viscosity and mobility. The core mechanism of CCHP technology lies in systematically altering the chemical composition of crude oil to achieve physical lightening. Experimental data show that as the huff-and-puff process proceeds, the saturate content in the produced oil continuously increases, while heavy components such as resins and asphaltenes decrease significantly to below 10%. This compositional improvement directly leads to an orders-of-magnitude reduction in crude viscosity (from >2000 mPa·s to below 200 mPa·s), substantially enhancing fluid mobility, which is the fundamental driver behind the improved cyclic and cumulative recovery.

This mechanism is strongly corroborated by its performance under different operational parameters. At the optimal post-depletion pressure (3.0 MPa), the system avoids the drawback of excessive early dissolved gas production associated with higher pressures, allowing the SARA composition to evolve steadily and continuously toward lighter fractions, thereby supporting high recovery. In contrast, under excessively high pressure (e.g., 6.0 MPa), the anomalous initial production of light components (saturates) followed by a sharp decline in subsequent cycles correlates directly with anomalous fluctuations in recovery and viscosity, underscoring the importance of stable compositional evolution for sustaining production.

Similarly, an optimized gas–liquid ratio (1.3 m^3^/m^3^) and chemical concentration (3% foam stabilizer + 1% foaming agent) enhance this process by generating stable secondary foam oil. An efficient foam system retards gas escape, allowing dissolved gas to more effectively extract and carry heavy components, ultimately driving recovery to its peak. This effect is observable both microscopically as a slowing decline in resin and asphaltene content, and macroscopically in crude oil viscosity, demonstrating the feedback effect of recovery dynamics on oil properties.

In summary, through the regulation of operational parameters, CCHP technology establishes optimal conditions. Conducted on this basis, the process of the CCHP drives a stable lightening transformation within the crude oil, centered on the rearrangement of SARA fractions. This transformation directly reduces viscosity and improves mobility, ultimately enhancing oil recovery. Elucidating this complete pathway, from microscopic composition to macroscopic performance, not only clarifies the core oil-producing process of the technology but also lays a solid theoretical foundation for its application to other heavy oil reservoirs with similar compositional characteristics.

### 3.5. Characteristics of Foamy Oil and Foam System

**Figure 16:**
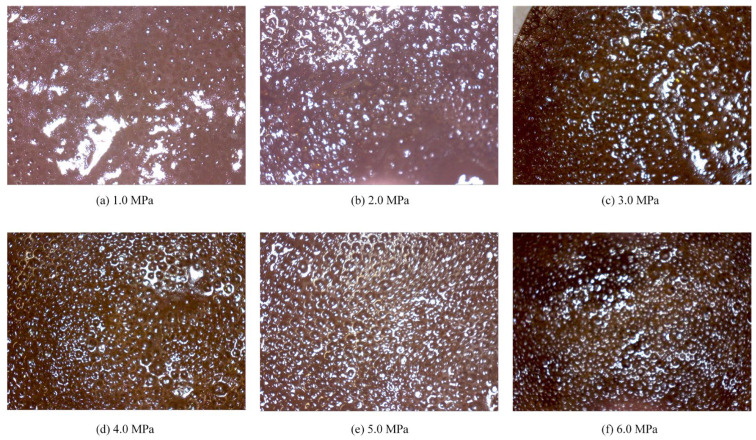
Morphology of foamy oil produced under various depletion pressures.

A lower post-depletion pressure results in less gas dissolved in the crude oil and a less-pronounced foamy oil effect. Figure 16 shows the morphology of foamy oil produced at various depletion pressures. As shown in Figure 16, at a post-depletion pressure of 6.0 MPa, the produced oil exhibits uniformly and densely distributes gas bubbles, which significantly enhances the fluidity of the crude oil. As the depletion pressure decreases, the density and uniformity of the bubbles gradually decline, with a noticeable difference observed below 3.0 MPa. The foamy oil effect diminishes markedly.

The dissolved gas in the crude oil manifested as dispersed gas bubbles, which effectively reduced the overall viscosity and density of the crude oil, thereby decreasing the viscous resistance experienced during flow. This foamy oil effect substantially improved the mobility of the crude oil and enhanced production performance.

**Figure 17:**
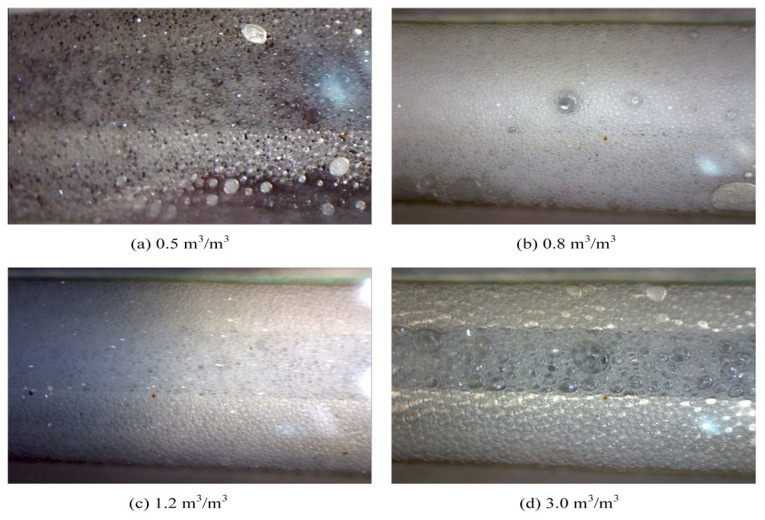
Flow behavior of foam generated for different gas–liquid ratios.

The gas–liquid ratio directly determines the lifespan and morphological evolution of foam. Figure 17 displays the flow behavior of foam generated under different gas–liquid ratios. From Figure 17, it can be seen that at a low gas–liquid ratio of 0.5 m^3^/m^3^, the liquid phase was excessive, resulting in poorly dispersed, discontinuous, and unstable foam. When the gas–liquid ratio increased to the optimal range of 0.8~1.2 m^3^/m^3^, the liquid was drained to the boundaries where bubbles intersect, causing the bubbles to be compressed into regular polyhedral shapes, and the foam became continuous and structurally stable at this stage, exhibiting optimal plugging performance. As it further increased to 3.0 m^3^/m^3^, the liquid film became excessively thin and ruptured, leading to intensified bubble coalescence. Consequently, enlarged bubble structures and reduced stability diminished the blocking capacity. Therefore, precise control of the gas–liquid ratio is essential for generating stable and high-performance foam.

**Figure 18:**
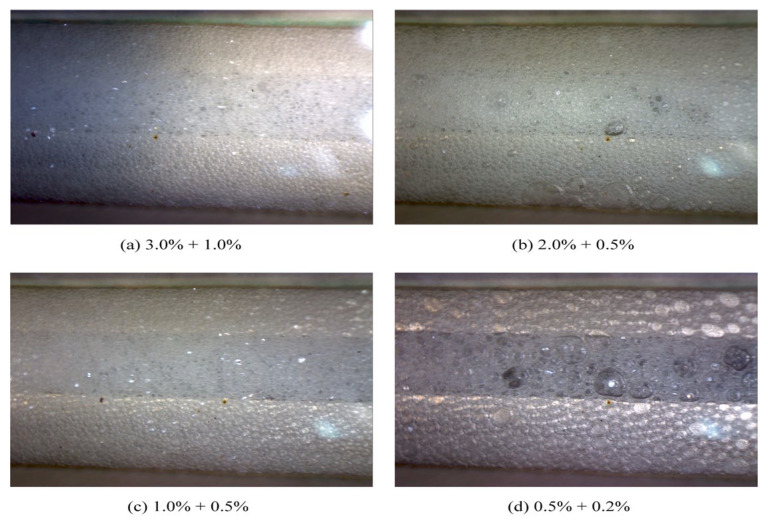
Flow behavior of foam generated for different chemical concentrations.

The concentrations of the foaming agent and the foam stabilizer jointly regulate the formation and decay processes of foam. Figure 18 indicates the flow behavior of foam generated under different chemical concentrations. As the chemical concentration decreased, foam coalescence caused by liquid film ruptured became notably apparent, resulting in a significant reduction in foam stability. This phenomenon was clearly observed when the concentration fell below 1.0% foaming agent with 0.5% foam stabilizer, under which conditions the plugging capacity of the foam was markedly weakened.

Insufficient foaming agent concentration led to excessively high surface tension in the system, hindering the effective generation of an adequate number of bubbles. Even if formed, the bubbles ruptured rapidly due to poor liquid film strength. Meanwhile, a deficiency in foam stabilizer concentration compromised the foam stability, reducing its structural integrity and shortening its lifespan. Therefore, achieving certain threshold concentrations of both chemicals is essential to synergistically create an ideal foam system characterized by fine bubbles, stable structure, and prolonged durability.

**Figure 19:**
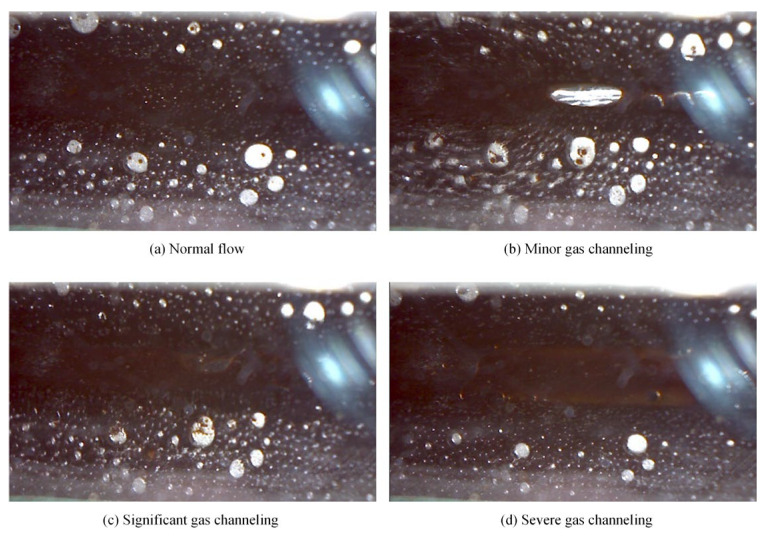
Gas channeling during oil production.

Gas channeling occurs as a consequence of the failure of the plugging system. Figure 19 illustrates the gas channeling during oil production. As is shown, flow pathways were formed due to extensive gas production resulting from gas channeling, and this phenomenon grew more evident as the condition became more severe.

Gas channeling frequently occurred in scenarios with high gas–liquid ratios and low chemical concentrations. The former is the most common cause of gas channeling, which led to thinning of the foam liquid films, a sharp decline in strength, and facilitate bubble coalescence and coarsening, ultimately resulting in the collapse into free gas. In the latter case, insufficient foaming agent concentration failed to effectively reduce the surface tension of the aqueous phase, resulting in poor foam generation. Meanwhile, inadequate foam stabilizer concentration compromised the enhancement of liquid film viscoelasticity and mechanical strength, making it impossible to maintain stability in high-salinity oil-bearing environments.

**Figure 20:**
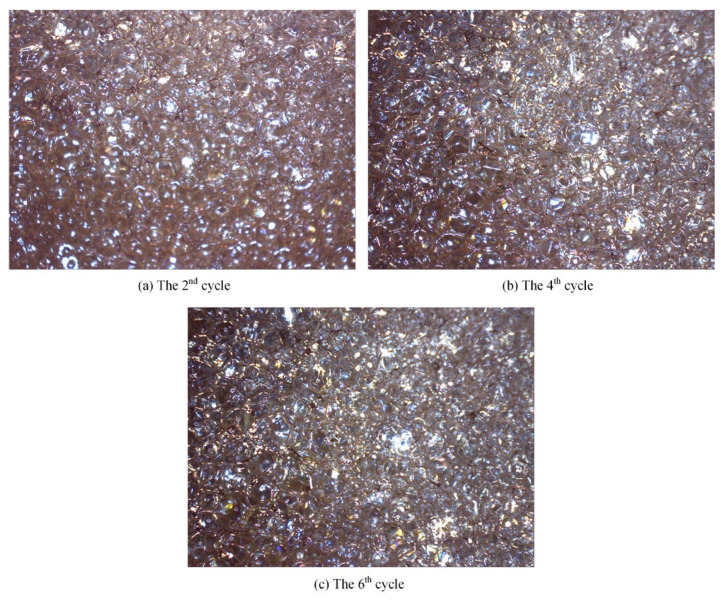
Morphology of foam under 10× magnification.

The morphology of foam produced in different cycles undergoes dynamic changes, which directly reflect the evolving conditions near the wellbore and the effectiveness of the foam treatment. Figure 20 displays the morphology of the foam under 10× magnification.

As observed across the production cycles, the foam structure transitioned from an oil-rich state to a homogeneous and stable configuration. In the 2nd cycle, the produced foam appeared brownish-yellow, with large and non-uniform bubble sizes. The foam walls were thick but weak, accompanied by visible oil droplets and oil films. By the 4th and 6th cycles, the produced foam exhibited light yellow to yellowish-white coloration, characterized by fine, uniform, and densely packed bubbles. The liquid films became thinner, yet more resilient, demonstrating enhanced stability and improved plugging capacity.

## 4. Application Potential, Limitations and Solutions

As an enhanced oil recovery technology, the CCHP involves the co-injection of natural gas and a stable foam system into the reservoir, integrating the dual advantages of replenishing reservoir energy, reducing oil viscosity and improving the sweep efficiency of natural gas and gas foam. The value of this technology is particularly pronounced in heterogeneous layered reservoirs. Its core mechanism lies in the ability of the foam to intelligently block high-permeability channels, thereby diverting subsequently injected methane gas into low-permeability oil-bearing layers, which significantly expands the sweep volume.

However, the CCHP does face challenges related to wellbore issues. Firstly, pressure drawdown operations during production and the extractive effect of CH_4_ on crude oil can lead to the precipitation and deposition of asphaltenes in the wellbore and near-wellbore region. This increases seepage resistance during injection and production processes and severely impacts production performance. Addressing this issue requires a combination of prevention and mitigation strategies. Chemically, efficient asphaltene dispersants can be injected; these adsorb onto the surface of asphaltene particles, dispersing them and inhibiting deposition. From a process perspective, it is essential to optimize injection and production parameters and control the production pressure differential to avoid conditions that would be favorable for massive asphaltene deposition.

Secondly, some reservoir environments characterized by a high temperature, high pressure, high salinity, and high oil saturation impose certain operational constraints, including limitations on injection methods and the composition of the chemical system. Therefore, the continuous optimization of injection strategies is key to addressing these challenges. Meanwhile, there are also other effective solutions, including consistently enhancing the stable foam system to ensure its compatibility with the reservoir environment, along with flexibly adjusting injection locations and methods based on the dynamic distribution of remaining oil saturation.

Furthermore, methane is both a potent greenhouse gas and a flammable hazard. The CCHP captures potentially escaping methane for utilization and sequesters it within a subsurface circulation system, effectively preventing its direct release into the atmosphere. Meanwhile, it enhances oil recovery in existing fields and reduces the carbon intensity per barrel of oil produced, establishing a greener and low-carbon extraction method. However, the injection and production processes carry environmental and safety risks, including potential methane leaks and contamination from chemical agents. Therefore, it is essential to establish a comprehensive methane monitoring and repair system, explore low-toxicity and biodegradable chemicals, and deploy a corresponding groundwater monitoring network.

## 5. Conclusions

Based on comprehensive investigation and analysis, the following conclusions can be drawn.

(1) Compared with surfactants and inorganic nanoparticles, polymers demonstrate superior comprehensive foam stabilization performance. XTG and HPAM-20M exhibit exceptional oil tolerance and salt resistance, achieving optimal foam stability with a drainage half-life of 48 h and an initial foaming volume of 280 mL.
(2) Foam stabilizers with a single component cannot achieve balanced optimization of foam generation and stability. The polymer–surfactant composite of XTG-CBM-DA demonstrates enhanced performance in both its foaming capacity, with an initial foaming volume of 330 mL, and its foam stability, with a drainage half-life of 48 h, effectively improving the overall foam properties.
(3) Lower post-depletion pressure correlates with reduced cyclic oil production. The differential production efficacy primarily manifests during the high-pressure stage where dissolved gas exists in crude oil. As the gas–liquid ratio increases, the recovery factor first rises then declines, with an optimal ratio of 1.2 m^3^/m^3^. Increasing the concentration of chemical agents progressively enhances production performance; therefore, the early stages of the CCHP require high-concentration foaming systems, considering the formation of adsorption effects.
(4) The density and viscosity of the produced oil progressively decrease with increasing cycles in the CCHP. High-pressure injection yields a slightly higher density and viscosity than low-pressure injection. Both parameters initially increase then decrease with rising gas–liquid ratios, while declining consistently with reduced concentrations of stable foaming systems.
(5) Saturate content gradually increases with additional cycles in the CCHP, while the content of aromatics shows minor decline. The content of resins and asphaltenes decreases significantly. Injection pressure, gas–liquid ratio, and chemical concentrations all exhibit a negative correlation with the content of saturates but a positive correlation with the content of asphaltenes in produced oil.
(6) The stability and performance of foamy oil and foam systems during production critically depend on precise control of key parameters. Insufficient gas–liquid ratios or chemical concentrations will trigger gas channeling, while the foam structure transitions from an oil-rich state to a homogeneous and stable configuration during the CCHP process.

## Figures and Tables

**Table 1:** SARA variation in crude oil from the MPE3 block.

Oil from MPE3	Saturates	Aromatics	Resins	Asphaltenes
Mass fraction (%)	6.02	39.64	33.5	20.84

**Table 2:** Content of missing light components in crude oil.

Components	n-Pentane	Isopentane	n-Hexane	n-Heptane	n-Octane	n-Nonane
Mass fraction (%)	0.01	0.01	0.04	0.05	0.08	0.15

**Table 3:** Types of foam stabilizers with a single component and a composite.

No.	Foam Stabilizers	Abbreviation-Specification	Types
1	Polyacrylamide	HPAM-2M	Polymer
2	Polyacrylamide	HPAM-10M	Polymer
3	Polyacrylamide	HPAM-20M	Polymer
4	Polyacrylamide	HPAM-26M	Polymer
5	Polyacrylamide	HPAM-35M	Polymer
6	Dodecanol	DA	Surfactant
7	Hexadecanol	HA	Surfactant
8	Modified silicone resin polyether microemulsion	FM-550	Surfactant
9	Modified silicone resin polyether emulsion	FM-550 E2	Surfactant
10	Modified acrylic polymer	MP-3000S	Polymer
11	Acrylic polymer film-forming agent	MP-50L	Polymer
12	Xanthan gum	XTG	Polymer
13	Acrylic polymer (Carbomer)	CBM	Polymer
14	Hydroxypropyl methyl cellulose type II	HPMC	Polymer
15	Hydroxyethyl cellulose	HEC	Polymer
16	Polyethylene glycol	PEO1500	Polymer
17	Polyethylene glycol	PEO20000	Polymer
18	Polyvinylpyrrolidone	K30	Polymer
19	Nano-silica	5 nm	Inorganic nanoparticle
20	Nano-silica	30 nm	Inorganic nanoparticle
21	Xanthan gum and Dodecanol	XTG-DA	Polymer–surfactant
22	Xanthan gum, acrylic polymer, and Dodecanol	XTG-CBM-DA	Polymer–surfactant
23	Polyacrylamide and Dodecanol	HPAM-DA	Polymer–surfactant
24	Polyacrylamide, Acrylic polymer, and Dodecanol	HPAM-CBM-DA	Polymer–surfactant

**Table 4:** Design of different core experiments.

Schemes No.	1	2	3	4	5	6	7	8	9	10	11	12	13	14	15
Post-depletion pressure (MPa)	1.0	2.0	3.0	4.0	5.0	6.0	3.0	3.0	3.0	3.0	3.0	3.0	3.0	3.0	3.0
Production pressure in the CCHP (MPa)	3.0	3.0	3.0	3.0	3.0	3.0	3.0	3.0	3.0	3.0	3.0	3.0	3.0	3.0	3.0
Gas–liquid ratio (m^3^/m^3^)	1.2	1.2	1.2	1.2	1.2	1.2	0.5	0.8	3.0	1.2	1.2	1.2	1.2	1.2	1.2
Concentration of foaming agent (%)	3.0	3.0	3.0	3.0	3.0	3.0	3.0	3.0	3.0	3.0	2.0	2.0	1.5	1.0	0.5
Concentration of foam stabilizer (%)	1.0	1.0	1.0	1.0	1.0	1.0	1.0	1.0	1.0	0.5	1.0	0.5	0.5	0.5	0.2

**Table 5:** Core properties.

Schemes No.	1	2	3	4	5	6	7	8
Porosity (%)	42.1	43.3	42.5	41.1	42.9	43.8	42.2	41.6
Permeability (um^2^)	7.3	7.2	7.4	7.6	7.2	7.0	7.6	7.3
Original oil in place (m^3^/m^3^)	98.8	97.9	97.5	97.0	98.1	96.8	97.3	99.0
**Schemes No.**	**9**	**10**	**11**	**12**	**13**	**14**	**15**	
Porosity (%)	43.0	42.6	42.0	45.1	43.9	42.7	42.9	
Permeability (um^2^)	7.2	7.3	7.3	7.7	7.4	7.4	7.7	
Original oil in place (m^3^/m^3^)	97.5	97.1	98.0	98.5	96.3	98.2	98.0	

## Data Availability

Data are contained within the article.
